# Optimal adjuvant chemotherapy for resected pancreatic adenocarcinoma: a systematic review and network meta-analysis

**DOI:** 10.18632/oncotarget.19082

**Published:** 2017-07-07

**Authors:** Jian-Bo Xu, Bin Jiang, Ya Chen, Fu-Zhen Qi, Jian-Huai Zhang, Hang Yuan

**Affiliations:** ^1^ Department of Hepatobiliary Surgery, Huai’an First People's Hospital, Nanjing Medical University, Nanjing, China; ^2^ Department of Coloproctologic Surgery, Zhejiang Provincial People's Hospital, Hangzhou, China

**Keywords:** pancreatic cancer, surgery resection, adjuvant chemotherapy, toxic effect, network meta-analysis

## Abstract

Adjuvant chemotherapy improves survival in patients with resected pancreatic cancer but the optimal regimen remains unclear. We aim to compare all possible adjuvant chemotherapy in terms of overall survival and toxic effects. Pubmed, Trial registries and Cochrane library databases for randomized controlled trials were searched until November 2016. Thirteen trials were included for network analysis and the hazard ratios (HRs) for survival and odds ratios for toxic effects were assessed via Aggregate Data Drug Information System software. Only S-1 chemotherapy improved 1-year, 3-year and 5-year survival compared with observation (HR (95% CI): 3.94 (1.18–12.34); 4.08 (1.58–8.24) and 5.09 (1.16–29.83) respectively). Although not significant, gemcitabine plus uracil/tegafur was associated with poorer 1-year and 3-year survival compared with observation (HR (95% CI): 0.85 (0.16–4.03) and 0.86 (0.23–2.95)). Adding radiation to chemotherapy has no significant improvement in survival. S-1 and gemcitabine plus capecitabine are currently the most effective adjuvant therapies for pancreatic cancer. While S1 has only been validated in Asian people, higher toxicity is an issue for gemcitabine plus capecitabine.

## INTRODUCTION

Pancreatic cancer is the fourth leading cause of cancer-related death with a 5-year survival rate of less than 7% [1]. Despite the continuous progress and development of surgery, prognosis has hardly improved due to the high propensity of tumor relapse [2, 3]. Adjuvant treatment has been advocated to decrease tumor recurrence and prolong overall survival (OS) after surgery resection. Several major adjuvant treatments including pre-operative chemotherapy (neoadjuvant chemotherapy), chemotherapy, chemoradiation (CRT) and chemotherapy plus chemoradiation have been used for more than thirty years.

As compare to post-operative chemotherapy, neoadjuvant chemotherapy is not affected by surgery-related complications and has potential advantages to improve rates of R0 resection, but randomized trials did not show a significant benefit [4, 5]. Previous studies involving CRT showed no survival benefit following pancreatic resection but increased adverse effects [6–8]. Mounting evidence shows that adjuvant chemotherapy can improve overall OS and relapse-free survival (RFS) in postoperative patients with pancreatic cancer. However, various chemotherapy regimens exist and the outcomes of these regimens remain controversial.

Gastrointestinal Tumor Study Group (GITSG) firstly indicated a statistically significant improvement in survival with fluorouracil plus CRT [9]. Median survival time in fluorouracil plus CRT group was 20 months vs 11 months in the control group. The European Study Group for Pancreatic Cancer (ESPAC) [6] performed a 2 × 2 factorial trial to compare observation, CRT, fluorouracil, and CRT plus fluorouracil. As compared to no chemotherapy group, chemotherapy group demonstrated a significant survival advantage with median survival time of 19.7 months vs 14.0 months and hazard ratio (HR) for death of 0.66 (95% confidence interval (95% CI) 0·52–0·83). In the Charite Onkologie (CONKO)-001 trial [10, 11], patients were randomized to adjuvant gemcitabine or observation. The promising results suggested that adjuvant gemcitabine could increase OS as well as RFS. ESPAC-3 trial [12], a large multicenter controlled trial, included 1088 postoperative patients with pancreatic cancer that randomly assigned to gemcitabine (*n* = 537) or fluorouracil (*n* = 551). There was no significant difference in OS and RFS between two groups with HR 0.94 (95% CI: 0.81–1.08) and 0.96 (95% CI: 0.84–1.10).

Traditional meta-analysis is unable to assess the effect of more than two chemotherapeutic drugs. In 2013, a network meta-analysis [13] was conducted to solve this problem with indirect comparison via a common comparator when no head-to-head trial existed and combined the direct and indirect comparisons [14, 15]. But this analysis neglected the RFS in patients who undergone surgery resection and previous analytical results now need to be updated as some novel chemotherapy regimens especially S1 and capecitabine have been published in recent years.

In order to reach a relatively general conclusion, we performed a network meta-analysis to compare the efficacy of different adjuvant chemotherapy in terms of 1-year, 3-year and 5-year survival for pancreatic adenocarcinoma patients.

## RESULTS

### Study characteristics and quality assessment

**Figure 1:**
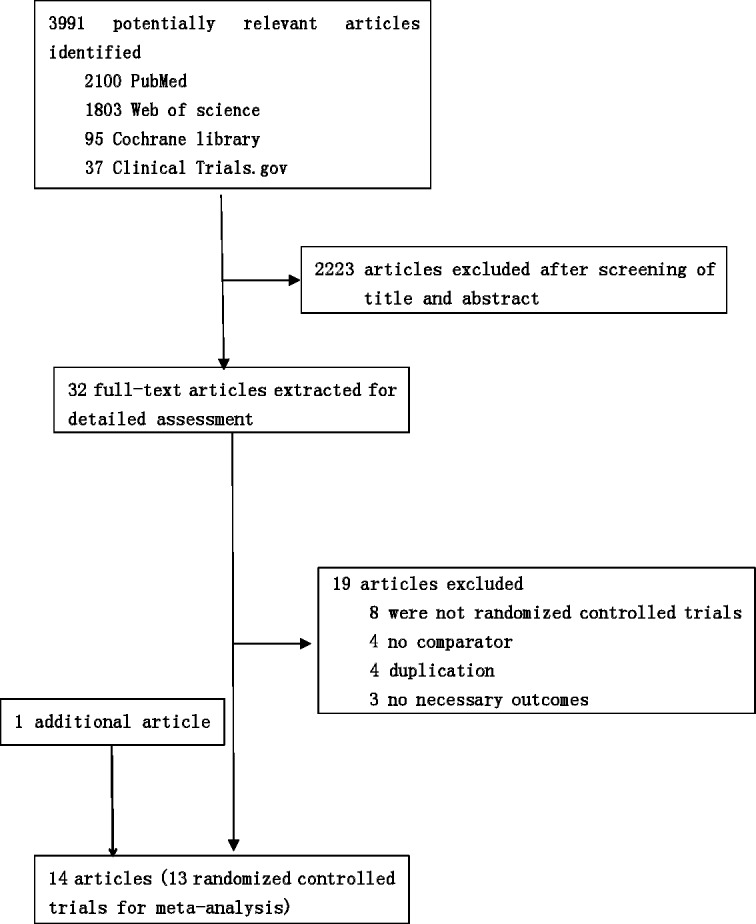
Literature search and selection

**Figure 2:**
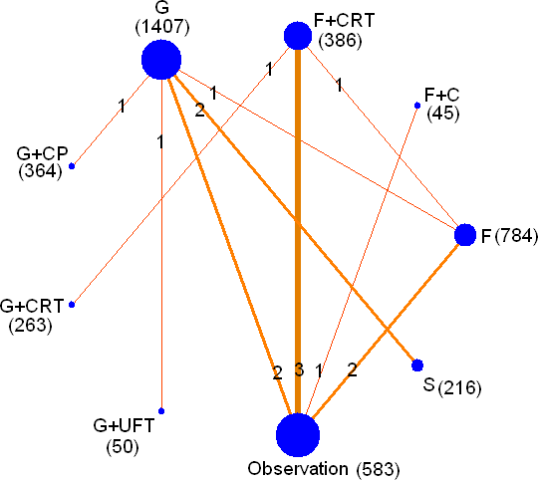
Network of the comparisons for the network meta-analysis The size of the nodes is proportional to the number of patients (in parentheses) to receive the treatment. The width of the lines is proportional to the number of trials (in the line) comparing the connected treatments.

We identified 4035 studies for review of title and abstract (Figure 1). After initial screening, 32 potentially eligible articles for full text were retrieved. Eight articles from the same research centre and the same author at different follow up, the early four reports were removed [6, 10, 16, 17]. Fourteen trials were included for systematic review. One article was excluded for network meta-analysis due to insufficient data [18]. As a result, 13 studies [7, 9, 11, 12, 19–27] including 4098 patients and nine regimens were involved in this network meta-analysis (Figure 2).

A total of 9 chemotherapy regimens including S-1, fluorouracil(F), gemcitabine(G), G plus capecitabine(CP), fluorouracil plus CRT, gemcitabine plus CRT, cisplatin plus epirubicin plus fluorouracil plus gemcitabine plus CRT (PEEG+CRT), gemcitabine plus uracil/tegafur (G+UFT) and fluorouracil plus cisplatin (F+C) were involved. 1407 patients received adjuvant gemcitabine, 784 were treated with fluorouracil and 216 were undergone S-1. Seven articles of surgery alone, four articles of F plus CRT and one article of G plus CRT, G plus CP, G plus uracil/tegafur and F plus cisplatin were reported (Table 1).

**Table 1:** Characteristics of included studies

Author (year)	Country	Registration number	Treatment /Control	Patients	Age^≠^	Man^#^ (%)	Overall survival (months/number)				Relapse–free survival (months)		Toxic effects
							Median time (95%CI)	1–year	3–year	5–year	Median time (95% CI)	HR (95% CI)	
Neoptolems JP (2017)	Many countries	ESPAC–4	G	336	65	63	25.5 (22.7–27.9)	292	79	9	13.1 (11.6–15.3)	1	196
			G + CP	364	65	55	28.0 (23.5–31.5)	302	102	19	13.9 (12.1–16.6)	0.86 (0.73–1.02)	226
Uesaka K (2016)	Japan	UMIN 000000655	S	187	66	57	46.5 (37.8–63.7)	172	111	80	22.9 (17.4–30.6)	0.6 (0.47–0.76)	26*
			G	190	66	54	25.5 (22.5–29.6)	151	73	45	11.3 (9.7–13.6)	1	138*
Shimoda M (2015)	Japan	NA	S	29	65*	28	21.5 (14.4–42.3)	25	4	NA	14.6 (8.8–28.4)	0.67 (0.4–1.11)	6*
			G	28	65*	43	18.0 (13.3–42.8)	21	4	NA	10.5 (7.0–28.4)	1	15*
Oettle H (2013)	Germany /Austria	CONK O–001	G	179	62	41	22.8	128	36	14	13.4 (11.6–15.3)	0.55 (0.44–0.69)	5*
			Observation	175	62	44	20.2	126	25	8	6.7 (6.0–7.5)	1	0
Reni M (2012)	Italy	NCT 00960284	G + CRT	42	61	79	26.2 (17.4–37.4)	NA	NA	NA	11.7 (7.0–20.5)	1	9*
			PEFG + CRT	38	60	63	31.6 (17.6–42.2)	NA	NA	NA	15.2 (10.3–25.7)	0.79 (0.56–1.13)*	26*
Regine WF (2011)	America	NCT 00003216	F + CRT	230	62	60	17.1	160	52	43	NA	NA	143
			G + CRT	221	61	53	20.5	155	59	41	NA	NA	175
Neoptolems JP (2010)	17 countries	NCT 00058201	F	551	63	55	23.0 (21.1–25)	413	109	15	14.1 (12.5–15.3)	1	379*
			G	537	63	55	23.6 (21.4–26.4)	415	103	13	14.3 (13.5–15.6)	0.96 (0.84–1.10)	221*
UenoH (2009)	Japan	NA	G	58	65	69	22.3 (16.1–30.7)	45	17	8	11.4 (8.0–14.5)	0.6 (0.40–0.89)	51*
			Observation	60	64	67	18.4 (15.1–25.3)	45	14	3	5.0 (3.7–8.9)	1	0
Neoptolems JP (2009)	many countries	ESPAC–1 +, ESPAC–3 v1	F	158	NA	NA	1 + :24.0 (18.8–29.4);3v1:25.9 (18.3– 36.3)	122	49	23	NA	NA	NA
			Observation	156	NA	NA	1 + :12.8 (10.2–16.9);3v1:20.3 (18.1–31.7)	94	29	15	NA	NA	NA
Yoshitomi H (2008)	Japan	NA	G	49	63	18	29.8	42	11	NA	12	1	15
			G + UFT	50	63	17	21.2	39	7	NA	12.3	0.97 (0.93–1.49)*	12
Smeenk HG (2007)	European countries	EORTC 40891	F + CRT	63	NA	NA	15.6 (13.2–21.6)	47*	15*	10*	18 (12–21.6)	0.81 (0.55–1.17)	10*
			Observation	57	NA	NA	12 (9.6–16.8)	29*	9*	4*	14.4 (10.8–20.4)	1	0
Kosuge T (2006)	Japan	NA	F + C	45	60	29	12.5	23	11	7	10.2	0.83 (0.56–1.23)	9
			Observation	44	60.1	21	15.8	29	10	4	8.6	1	0
Neoptolemos JP (2004)	European countries	ESPAC–1 trial	Observation	69	NA	NA	16.9 (12.3–24.8)	39*	15*	4*	NA	NA	0
			F	75	NA	NA	21.6 (13.5–27.3)	55*	21*	9*	NA	NA	11
			F + CRT	72	NA	NA	19.9 (14.2–22.5)	44*	17*	2*	NA	NA	16
KalserMH (1985)	America	NA	F + CRT	21	NA	NA	20	14*	5*	3*	NA	0.6 (0.43–0.85)*	3
			Observation	22	NA	NA	11	11*	5*	2*	NA	1	0

S, S-1; F, fluorouracil; G, gemcitabine, CRT, chemoradiation; C, Cisplatin; UFT, uracil/tegafur; NA, not available;^≠^ mean age; ^#,^percentage of male;*estimated from summary statistics.

**Figure 3:**
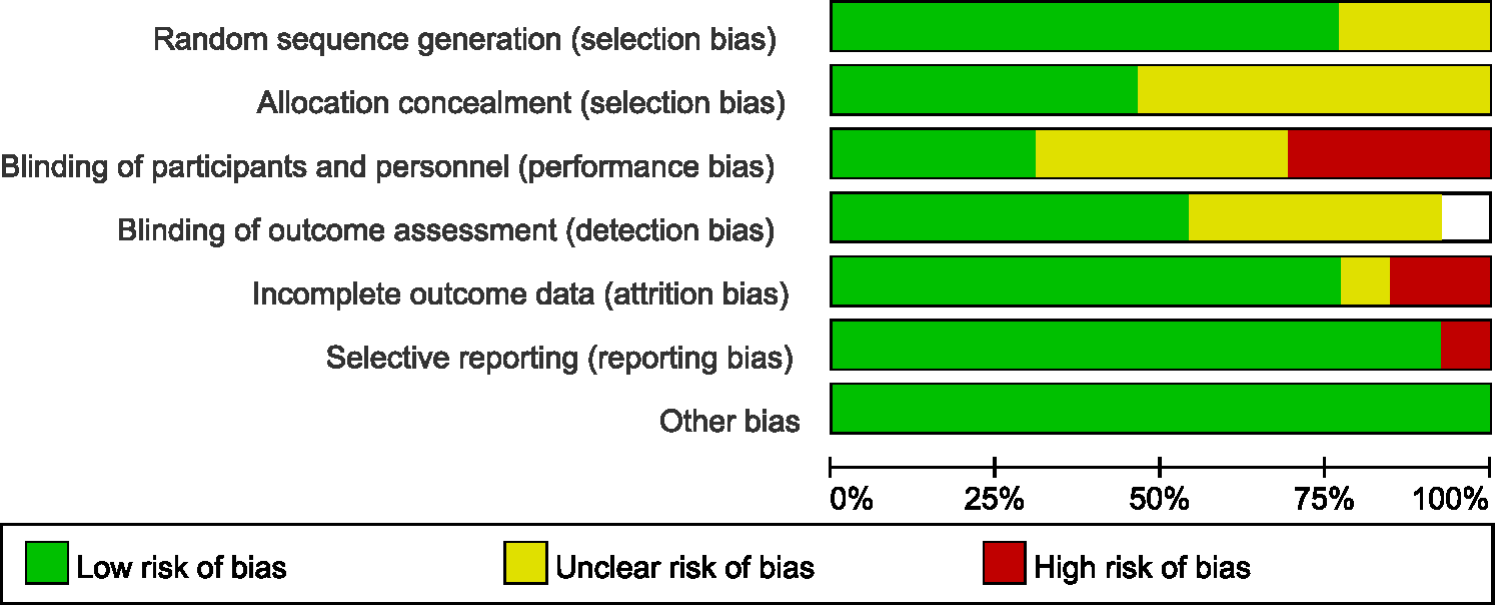
Cochrane risk of bias tool results

The most common method of randomization was random numbers generated from computer or a minimization technique. The blinding method was not performed in all trials, but the outcome was not likely to be influenced by lack of blinding in some articles. The included studies seemed to be at low risk of bias by the Cochrane Risk of Bias tool (Figure 3).

### Efficacy of adjuvant chemotherapy

**Figure 4:**
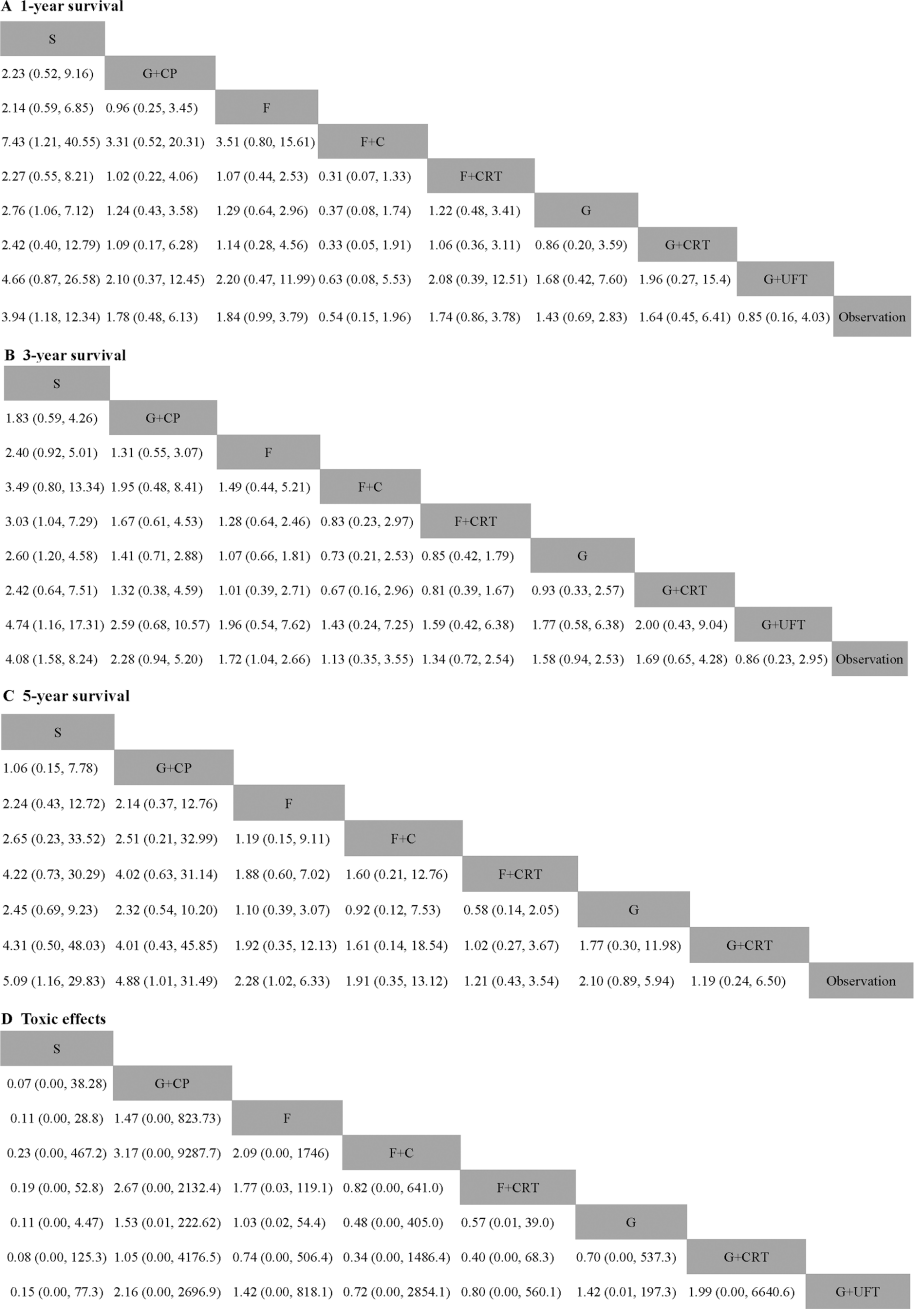
Pooled hazard ratios for survival and pooled odds ratios for overall toxic effects (**A**) 1-year survival; (**B**) 3-year survival; (**C**) 5-year survival; (**D**) Overall grade 3–4 toxic effects. The column treatment is compared with the row treatment. Numbers in parentheses indicate hazard rations and 95% credible intervals.

Thirteen studies reported information on 1-year and 3-year survival. Eleven trials involved data on 5-year survival. For 1-year survival, only adjuvant S-1 showed a significant effect compared with observation (HR 3.94, 95% CI 1.18–12.34). Fluorouracil plus chemoradiation did not provide a survival benefit over fluorouracil (HR 1.07, 95% CI 0.80–15.61). As compared with observation, most chemotherapy had a tendency of survival benefit, whereas adjuvant G+UFT and F+C provided a poorer survival tendency (HR 0.85, 95% CI 0.16–4.03 and HR 0.54, 95% CI 0.15–1.96 respectively) (Figure 4A).

For 3-year survival, both S and F demonstrated a significant higher survival rate than observation (HR 4.08, 95% CI 1.58–8.24 and HR 1.72, 95% CI 1.04–2.66). Both G plus CP and F did not provide survival benefit over S with HR (95% CI) 1.83 (0.59–4.26) and 2.40 (0.92–5.01). Although not significant, G+UFT was associated with worse survival compared with observation (HR0.86, 95% CI 0.23–2.95). The result showed non-inferiority of gemcitabine and fluorouracil to chemoradiation plus gemcitabine and chemoradiation plus fluorouracil (HR0.93, 95% CI 0.33–2.57 and HR 1.28, 95% CI 0.64–2.46 respectively) (Figure 4B).

S, G plus CP and F were associated with higher 5-year survival rate than observation (HR (95% CI): 5.09 (1.16–29.83), 4.88 (1.01–31.49) and (2.28, 1.02–6.33 respectively). Gemcitabine and fluorouracil was slightly more effective compared with chemoradiation plus gemcitabine and chemoradiation plus fluorouracil (HR 1.77, 95% CI 0.30–11.98 and HR 1.88, 95% CI 0.60–7.02 respectively) (Figure 4C).

We did not performed network analysis for RFS due to the limited number of direct comparative studies. But RFS rates were significantly higher in the S-1 groups than that in G groups (22.9 months vs 11.3 months, HR 0.6, 95% CI 0.47–0.76). Longer, although not significant, RFS than G+CRT was also noted with PEEG+CRT.

### Adverse effects

Common side effects are dose-dependent including neutrophil, leucocyte, haemoglobin, platelet levels, fatigue, anorexia and diarrhoea. Some articles reported the number of grade 3–4 anaemia, thrombocytopenia, leucopenia, anorexia and fatigue toxic effects separately (Table 1). We speculated the overall toxic effects with the largest of these numbers. There was a tendency that gemcitabine plus capecitabine increased toxic effects. Chemoradiation plus gemcitabine was associated with more frequent toxic effects compared with gemcitabine, but this did not reach statistical difference (HR 0.70, 95% CI 0.00, 537.3) (Figure 4D).

### Rank test

**Figure 5:**
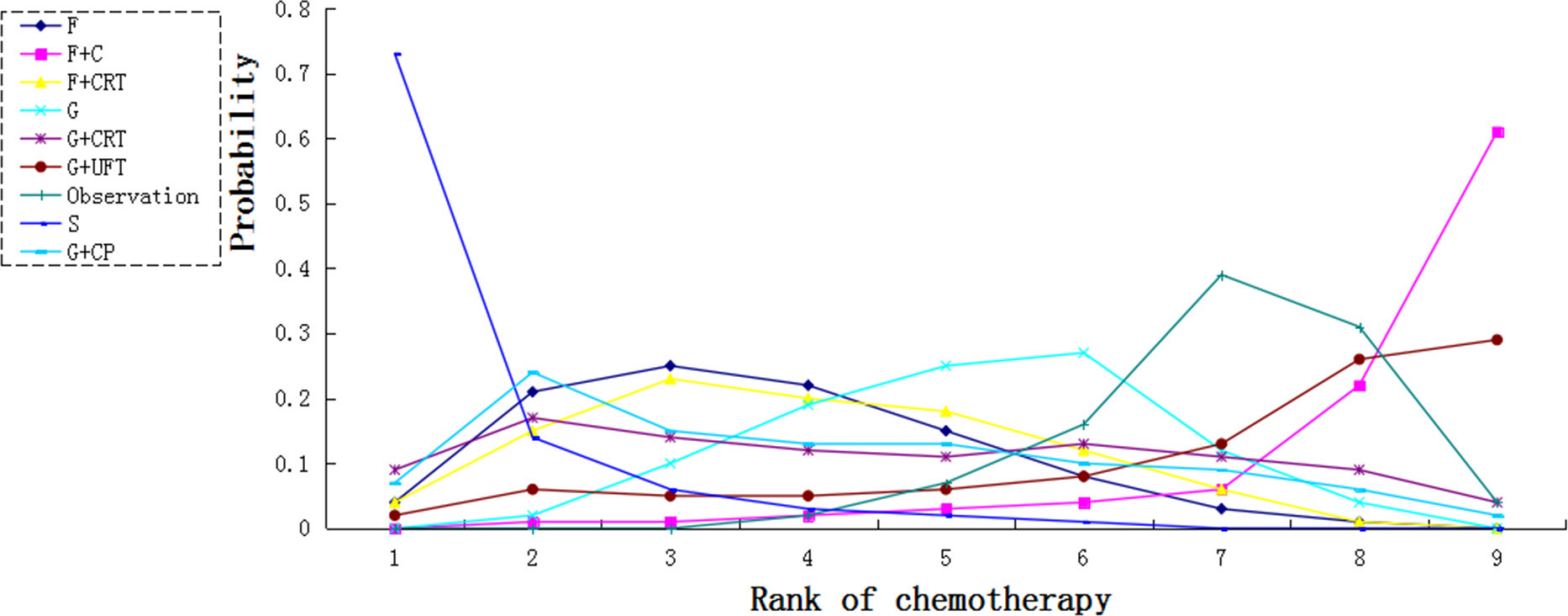
Ranking for 1-year survival of 8 chemotherapy regimens Rank 1 is best and rank N is worst.

**Figure 6:**
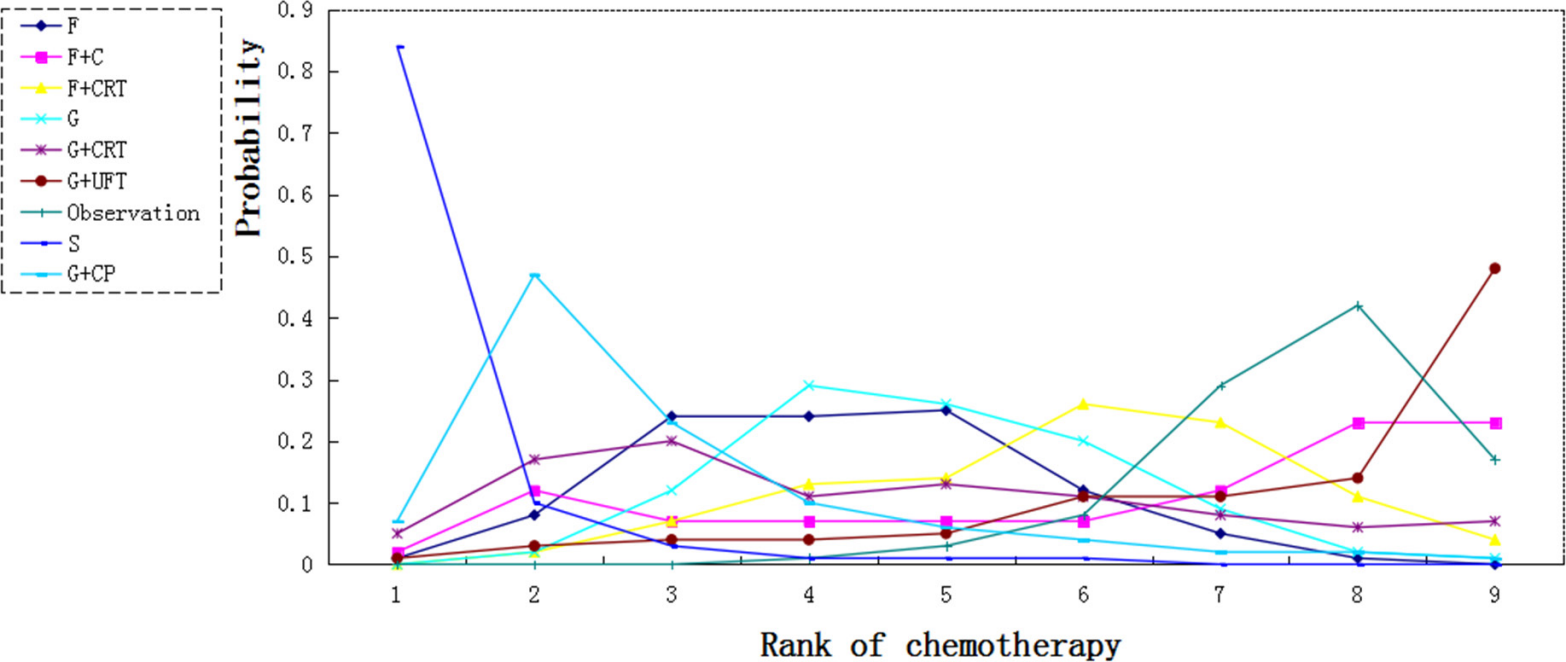
Ranking for 3-year survival of 8 chemotherapy regimens Rank 1 is best and rank N is worst.

**Figure 7:**
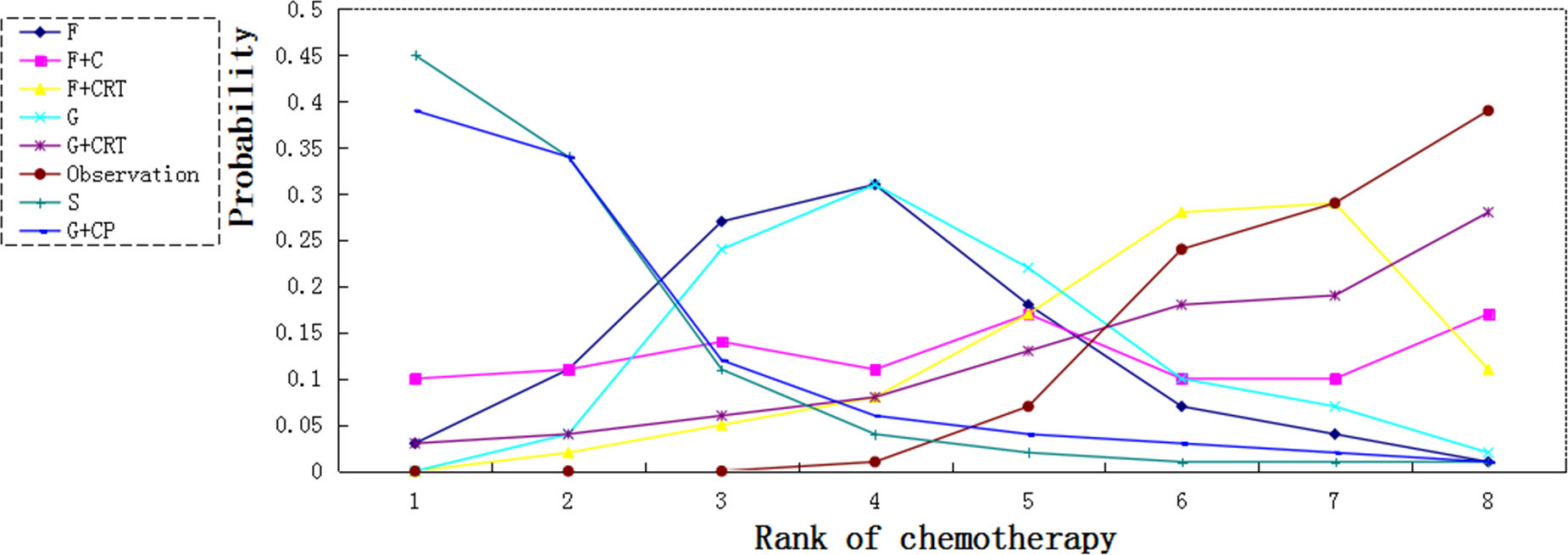
Ranking for 5-year survival of 7 chemotherapy regimens Rank 1 is best and rank N is worst.

**Figure 8:**
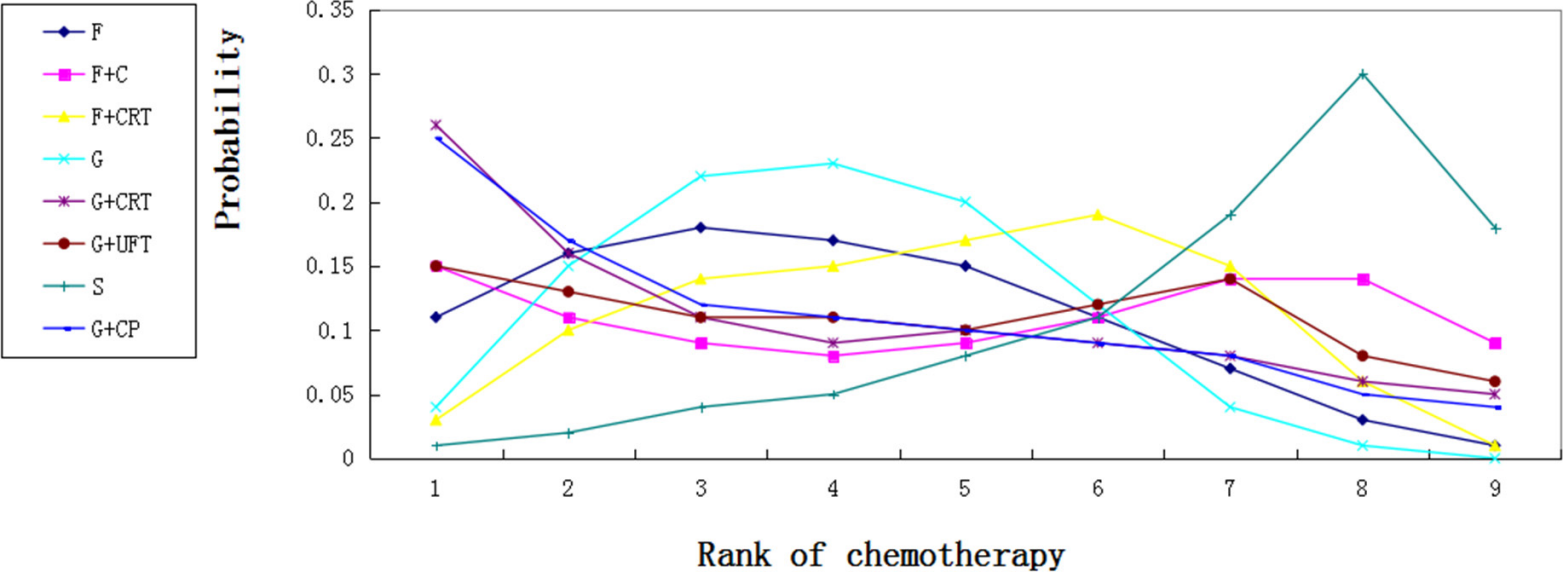
Ranking for toxic effects of 8 chemotherapy regimens Rank 1 is worst and rank N is best.

We calculated probabilities of best chemotherapy for each intervention at overall survival (Figures 5–7). Obviously, S-1 ranked best to prolong overall survival in term of 1- and 3-year. S-1 and gemcitabine plus capecitabine were the best efficacious chemotherapy in prolonging 5-year overall survival. F + C might rank worst probability of improving OS at 1-year and 3-year. Similar ranking of chemoradiation plus gemcitabine, fluorouracil and gemcitabine was showed. S-1 chemotherapy ranked the least toxic, whereas gemcitabine plus capecitabine ranked the most toxic (Figure 8).

### Node split of network meta-analysis

Pair wise meta-analysis did not suggest inconsistency in direct comparison (data not shown). The node-splitting models in our network meta-analysis included F, F+CRT; F, G, F, observation and G, observation. The node-splitting models suggested that there was no statistical difference between direct and indirect comparisons (*P* > 0.05) (Table 2).

**Table 2:** Results of node-splitting models for the test of difference between direct and indirect effect in the analysis of overall survival and toxic effects

Survival	Name	Direct effect	Idirect effect	Overall	*P*
1-year survival	F, F + CRT	−0.48 (−1.66,0.60)	0.47 (−0.68,1.58)	−0.06 (−0.93,0.83)	0.19
	F, G	0.13 (−0.69,0.90)	−0.84 (−1.78,0.07)	−0.25 (−1.09,0.45)	0.08
	F, OBS	−0.72 (−1.65,0.15)	−0.53 (−1.77,0.48)	−0.61 (−1.33,0.01)	0.73
	G, OBS	−0.03 (−0.73,0.65)	−1.01 (−2.06,0.01)	−0.36 (−1.04,0.38)	0.10
3-year survival	F, F + CRT	−0.33 (−1.22,0.69)	−0.07 (−1.14,0.91)	−0.25 (−0.90,0.45)	0.74
	F, G	−0.04 (−0.86,0.76)	−0.20 (−1.12,0.75)	−0.07 (−0.59,0.41)	0.8
	F, OBS	−0.50 (−1.19,0.07)	−0.44 (−1.28,0.24)	−0.54 (−0.98,−0.04)	0.81
	G, OBS	−0.38 (−1.09,0.27)	−0.55 (−1.52,0.47)	−0.46 (−0.93,0.06)	0.87
5-year survival	F, F + CRT	−0.33 (−1.22,0.69)	−0.07 (−1.14,0.91)	−0.25 (−0.90,0.45)	0.74
	F, G	−0.04 (−0.86,0.76)	−0.20 (−1.12,0.75)	−0.07 (−0.59,0.41)	0.78
	F, OBS	−0.50 (−1.19,0.07)	−0.44 (−1.28,−0.24)	−0.54 (−0.98,−0.04)	0.81
	G, OBS	−0.38 (−1.09,0.27)	−0.55 (−1.52,0.47)	−0.46 (−0.93,0.06)	0.87
Toxic effect	F, F + CRT	0.43 (−4.92,5.74)	−2.13 (−7.97,4.30)	−0.63 (−4.85,3.48)	0.43
	F, G	−1.16 (−5.83,3.56)	2.56 (−4.01,7.67)	−0.07, (−3.86,3.82)	0.25
	F, OBS	−3.00 (−9.28,3.10)	−3.81 (−8.64,1.25)	−3.60 (−7.43,0.33)	0.82
	G, OBS	−4.84 (−8.32,−0.97)	−1.16 (−7.22,5.03)	−3.65 (−7.04,−0.15)	0.23

S, S-1; F, fluorouracil; G, gemcitabine, CRT, chemoradiation; C, Cisplatin; UFT, uracil/tegafur; NA, not available; *estimated from summary statistics.

## DISCUSSION

The objective of this network meta-analysis was to investigate the optimal chemotherapy in patients with pancreatic adenocarcinoma after resection. We suggest that adjuvant S-1 chemotherapy improves overall survival compare with other remaining chemotherapy regimens but not increases toxic effects.

Network meta-analysis has been widely accepted by clinical researchers and International Health Technology Assessment Organizations with solid statistical foundation. Unlike traditional meta analysis, network meta analysis compares multiple interventions simultaneously, combines direct and indirect comparisons and ranks these interventions according to their efficacy with probabilities. Moreover, despite of no significant difference among all interventions, network analysis could screen the best one or two interventions according to the ranking of possibilities.

Our results showed the HR for 5-year survival was 2.45 and 2.24 in S-1 group in comparison to gemcitabine and fluorouracil. Neoptolemos JP revealed [12] no significant inferiority of fluorouracil in OS and RFS to gemcitabine (HR 0.94, 95% CI 0.81–1.08 and HR 0.96, 95% CI 0.84–1.10). S-1 is a novel oral drug containing tegafur, gimeracil and oteracil which has been widely used for treatment of many types of advanced cancer [28–30]. Geimidine has a strong and effective dihydropyrimidine dehydrogenase (DPD) selective antagonistic effect, thus inhibit 5-FU catabolism, maintain high concentration of fluorouracil in tumor tissue and enhance antitumor effect of fluorouracil. The GEST Study [27] demonstrated that S-1 was slightly more effective in advanced or metastatic pancreatic cancer in terms of OS compared with gemcitabine (median OS 9.7 vs 8.8 months, HR 0.96, 95% CI 0.78–1.18). However, JASPAC-01 trial showed a significant superiority of S-1 to gemcitabine in OS (HR 0·57, 95% CI 0·44–0·72). They speculated that DPD inhibitory oral fluoropyrimidine including S-1 and uracil/tegafur might be more effective in the adjuvant setting. Our results do not support this review. We revealed that adjuvant G+UFT did not improve OS compared with observation. The possible reason of improvement of OS in S-1 might be a higher response rate of S-1 than gemcitabine [31]. Another possible reason might be that oral chemotherapy is more acceptable to patients compare with intravenous chemotherapy. The HRs for 5-year survival were 5.09 (1.16, 29.83) for S-1 and 4.88 (1.01, 31.49) for gemcitabine plus capecitabine compared with observation and the two chemotherapy have similar ranking for 5-year survival. However, the HRs for 1-, 3-year survival in S-1 were higher than in gemcitabine plus capecitabine. The main reason for this difference is that 69% of the patients in JASPAC-1 trial had a R0 performance status compared with 42% in ESPAC-4 trial, and 37% had an N0 status in JASPAC-1 compared with 20% in ESPAC-4.

Adjuvant chemoradiation plus gemcitabine, fluorouracil and gemcitabine had similar ranking in survival. Chemoradiation plus fluorouracil did not provide survival benefit compared with fluorouracil. Chemoradiation plus gemcitabine increased toxic effects compared with gemcitabine (HR0.70, 95% CI 0.00, 537.3). Thus, fluorouracil or gemcitabine were more favourable than chemoradiation plus fluorouracil or gemcitabine in terms of the balance between survival benefit and toxic effects.

Our meta-analysis estimates every chemotherapy regimen individually and compares all drugs simultaneously. In 2013, a network meta-analysis was conducted to assess the optimal treatment for resected pancreatic cancer. We updated this analysis and our study also has several strengths. The previous analysis reviewed major adjuvant treatments including chemotherapy and chemoradioation. The results of their analysis showed that CRT did not provide survival benefit but increased toxic effects. Thus, we systematically reviewed all possible chemotherapy (9 regimens) for postoperative pancreatic cancer rather than focusing on only several major chemotherapy drugs. Furthermore, we also reviewed RFS in patients with pancreatic cancer after surgery and draw the rank probability plot to find out which chemotherapy is the best. Median RFS was longer in S-1 group than that in other chemotherapy groups. We suggest that S-1 prolongs overall survival and postpones recurrence and is the optimum treatment regimen.

Major toxic events (grade 3 or 4) available in the S-1 group were abnormal neutrophil, haemoglobin, leucocyte, platelet levels, anorexia, fatigue and diarrhoea. Adjuvant S-1 was associated with less frequent grade 3 or 4 leucopenia, neutropenia, but with more frequent stomatitis and diarrhoea. JASPAC-01 trial showed that 48 (25%) patients in the gemcitabine group and 40 (21%) in the S-1 group stopped treatment before completion due to toxic events; In ESPAC-4 trial, 52 (41%) patients in gemcitabine group and 79 (47%) in gemcitabine plus capecitabine group discontinued treatment because of adverse events. Gemcitabine plus capecitabine was associated with higher frequently toxic events compared with other chemotherapy. Consistent with previous study, our results showed that chemoradiation plus gemcitabine ranked the worst toxic. However, toxic effects in our network analysis need be interpreted with some caution because some articles did not report the overall number of toxic effects.

Some limitations exist in our analysis. A major limitation of our study is that all patients treated with S-1 in our study were from Japan. Difference of pharmacokinetics and pharmacodynamics for S-1 between Westerners and Asians makes it uncertainty to apply our results to European and North American patients [32]. Second, meta-analysis was not conducted on RFS due to insufficient outcome. Third, the sizes were small in patients treated with gemcitabine plus uracil/tegafur and fluorouracil plus cisplatin.

In conclusion, our network meta-analysis suggest that S-1 is the optimum adjuvant chemotherapy for both short and long term survivals in Asian patients with pancreatic cancer after resection, this results need to be assessed in non-Asian patients. Gemcitabine plus capecitabine also has promising outcomes for these patients accompanied with high toxic events. Chemotherapy plus fluorouracil or gemcitabine is inferior to fluorouracil or gemcitabine alone in terms of overall survival and toxic effects.

## MATERIALS AND METHODS

### Search strategy

Systematic review was conducted on the base of PRISMA (Preferred Reporting Items for Systematic Reviews and Meta-Analyses) guidelines [33]. An electronic search of Pubmed, Web of science, Cochrane library databases and Clinical Trials.gov was performed for randomised controlled trials (RCTs) with key words “pancreatic cancer (adenocarcinoma) and adjuvant chemotherapy” until November 2016 in English. We also manually searched reference lists of published articles and included eligible trials in previous meta-analysis [13, 34].

### Criteria for inclusion and exclusion

Inclusion criteria for this study were as follows: (i) all patients were histologically proven pancreatic ductal adenocarcinoma with macroscopically curative resection; (ii) trials were RCTs and patients received adjuvant chemotherapy or chemotherapy plus CRT; (iii) reports mentioned at least one of the outcomes of 1-year, 3-year and 5-year survival rates.

Trials with the following conditions were excluded: (i) advanced or metastatic pancreatic cancer; (ii) non-randomized design or retrospective analysis; (iii) neoadjuvant therapy or adjuvant CRT.

### Data extraction

Three investigators (Jian-Bo Xu, Bin Jiang and Hang Yuan) independently reviewed the full manuscripts of eligible studies and extracted the data from each article including patient characteristics, treatment, registration number and outcomes (OS, RFS and overall grade 3–4 toxic effects). Any discrepancies among investigators were solved by selected quality items and discussion. We used Engauge Digitizer 4.1 to calculate necessary data when they were not report in the article.

### Risk of bias and data analysis

The quality of selected articles was independently assessed by two reviewers in accordance with the Cochrane Risk of Bias Tool including random sequence generation, allocation concealment of treatment, blinding, incomplete outcome data, selective outcome reporting and other sources of bias.

We performed the traditional pair-wise meta-analysis and network plot with STATA 12.0. Pair-wise and network meta-analysis was done by automated software Aggregate Data Drug Information System (ADDIS 1.16.8). This approach combines direct and indirect evidence considering pair of treatments in one joint analysis [35]. Indirect comparison between two treatments requires one common comparator. We used node split model to assess inconsistency between indirect and direct comparisons. The consistency model was used when the node split model was *P* > 0.05; otherwise, the inconsistency model was done. Treatments were ranked for each outcome based on their probabilities. We calculated the HRs for each treatment by comparing with other treatments sequentially. Chemotherapy regimens were ranked based on their posterior probabilities. Toxic effects analysis was done as described previously [13]. We also produced the rank probability plot to find out the best chemotherapy.

## Abbreviations

HR: hazard ratio
OS: overall survival
RFS: relapse-free survival
GITSG: Gastrointestinal Tumor Study Group
ESPAC: European Study Group for Pancreatic Cancer
CI: confidence index
DPD: dihydropyrimidine dehydrogenase
PRISMA: Preferred Reporting Items for Systematic Reviews and Meta-Analyses
RCT: randomized controlled trial
S: S-1
F: fluorouracil
G: gemcitabine
CRT: chemoradiation
C: Cisplatin
UFT: uracil/tegafur

## References

[1] Siegel R, Naishadham D, Jemal A (2012). Cancer Statistics, 2012. CA Cancer J Clin.

[2] Cress RD, Yin DX, Clarke L, Bold R, Holly EA (2006). Survival among patients with adenocarcinoma of the pancreas: A population-based study (United States). Cancer Causes Control.

[3] Alexakis N, Halloran C, Raraty M, Ghaneh P, Sutton R, Neoptolemos JP (2004). Current standards of surgery for pancreatic cancer. Br J Surg.

[4] Casadei R, Di Marco M, Ricci C, Santini D, Serra C, Calculli L, D'Ambra M, Guido A, Morselli-Labate AM, Minni F (2015). Neoadjuvant Chemoradiotherapy and Surgery Versus Surgery Alone in Resectable Pancreatic Cancer: A Single-Center Prospective, Randomized, Controlled Trial Which Failed to Achieve Accrual Targets. J Gastrointest Surg.

[5] Brunner TB, Witzigmann H, Marti L, Bechstein WO, Bruns C, Jungnickel H, Schreiber S, Grabenbauer GG, Meyer T, Merkel S, Fietkau R, Hohenberger W (2015). Neoadjuvant chemoradiation therapy with gemcitabine/cisplatin and surgery versus immediate surgery in resectable pancreatic cancer: results of the first prospective randomized phase II trial. Strahlenther Onkol.

[6] Neoptolemos JP, Dunn JA, Stocken DD, Almond J, Link K, Beger H, Bassi C, Falconi M, Pederzoli P, Dervenis C, Fernandez-Cruz L, Lacaine F, Pap A (2001). Adjuvant chemoradiotherapy and chemotherapy in resectable pancreatic cancer: a randomised controlled trial. Lancet.

[7] Neoptolemos JP, Stocken DD, Friess H, Bassi C, Dunn JA, Hickey H, Beger H, Fernandez-Cruz L, Dervenis C, Lacaine F, Falconi M, Pederzoli P, Pap A (2004). A randomized trial of chemoradiotherapy and chemotherapy after resection of pancreatic cancer. N Engl J Med.

[8] Liao WC, Chien KL, Lin YL, Wu MS, Lin JT, Wang HP, Tu YK (2013). Adjuvant treatments for resected pancreatic adenocarcinoma: a systematic review and network meta-analysis. Lancet Oncol.

[9] Kalser MH, Ellenberg SS (1985). Pancreatic cancer. Adjuvant combined radiation and chemotherapy following curative resection. Arch Surg.

[10] Oettle H, Post S, Neuhaus P, Gellert K, Langrehr J, Ridwelski K, Schramm H, Fahlke J, Zuelke C, Burkart C, Gutberlet K, Kettner E, Schmalenberg H (2007). Adjuvant chemotherapy with gemcitabine vs observation in patients undergoing curative-intent resection of pancreatic cancer: a randomized controlled trial. JAMA.

[11] Oettle H, Neuhaus P, Hochhaus A, Hartmann JT, Gellert K, Ridwelski K, Niedergethmann M, Zulke C, Fahlke J, Arning MB, Sinn M, Hinke A, Riess H (2013). Adjuvant chemotherapy with gemcitabine and long-term outcomes among patients with resected pancreatic cancer: the CONKO-001 randomized trial. JAMA.

[12] Neoptolemos JP, Stocken DD, Bassi C, Ghaneh P, Cunningham D, Goldstein D, Padbury R, Moore MJ, Gallinger S, Mariette C, Wente MN, Izbicki JR, Friess H (2010). Adjuvant chemotherapy with fluorouracil plus folinic acid vs gemcitabine following pancreatic cancer resection: a randomized controlled trial. JAMA.

[13] Liao WC, Chien KL, Lin YL, Wu MS, Lin JT, Wang HP, Tu YK (2013). Adjuvant treatments for resected pancreatic adenocarcinoma: a systematic review and network meta-analysis. Lancet Oncol.

[14] Song F, Altman DG, Glenny AM, Deeks JJ (2003). Validity of indirect comparison for estimating efficacy of competing interventions: empirical evidence from published meta-analyses. Br Med J.

[15] Sutton A, Ades AE, Cooper N, Abrams K (2008). Use of indirect and mixed treatment comparisons for technology assessment. Pharmacoeconomics.

[16] (1987). Further evidence of effective adjuvant combined radiation and chemotherapy following curative resection of pancreatic cancer. Gastrointestinal Tumor Study Group. Cancer.

[17] Regine WF, Winter KA, Abrams RA, Safran H, Hoffman JP, Konski A, Benson AB, Macdonald JS, Kudrimoti MR, Fromm ML, Haddock MG, Schaefer P, Willett CG (2008). Fluorouracil vs gemcitabine chemotherapy before and after fluorouracil-based chemoradiation following resection of pancreatic adenocarcinoma: a randomized controlled trial. JAMA.

[18] Reni M, Balzano G, Aprile G, Cereda S, Passoni P, Zerbi A, Tronconi MC, Milandri C, Saletti P, Rognone A, Fugazza C, Magli A, Di Muzio N (2012). Adjuvant PEFG (Cisplatin, Epirubicin, 5-Fluorouracil, Gemcitabine) or Gemcitabine Followed by Chemoradiation in Pancreatic Cancer: A Randomized Phase II Trial. Ann Surg Oncol.

[19] Kosuge T, Kiuchi T, Mukai K, Kakizoe T, Jsap (2006). A multicenter randomized controlled trial to evaluate the effect of adjuvant cisplatin and 5-fluorouracil therapy after curative resection in cases of pancreatic cancer. Jpn J Clin Oncol.

[20] Smeenk HG, van Eijck CH, Hop WC, Erdmann J, Tran KC, Debois M, van Cutsem E, van Dekken H, Klinkenbijl JH, Jeekel J (2007). Long-term survival and metastatic pattern of pancreatic and periampullary cancer after adjuvant chemoradiation or observation: long-term results of EORTC trial 40891. Ann Surg.

[21] Yoshitomi H, Togawa A, Kimura F, Ito H, Shimizu H, Yoshidome H, Otsuka M, Kato A, Nozawa S, Furukawa K, Miyazaki M (2008). A randomized phase II trial of adjuvant chemotherapy with uracil/tegafur and gemcitabine versus gemcitabine alone in patients with resected pancreatic cancer. Cancer.

[22] Neoptolemos JP, Fau Stocken Dd, Tudur Smith C, Tudur Smith C, Fau-Bassi C, Bassi C, Fau-Ghaneh P, Ghaneh P, Fau-Owen E, Owen E, Fau-Moore M, Moore M, Fau-Padbury R (2009). Adjuvant 5-fluorouracil and folinic acid vs observation for pancreatic cancer: composite data from the ESPAC-1 and -3(v1) trials. Br J Cancer.

[23] Ueno H, Kosuge T, Matsuyama Y, Yamamoto J, Nakao A, Egawa S, Doi R, Monden M, Hatori T, Tanaka M, Shimada M, Kanemitsu K (2009). A randomised phase III trial comparing gemcitabine with surgery-only in patients with resected pancreatic cancer: Japanese Study Group of Adjuvant Therapy for Pancreatic Cancer. Br J Cancer.

[24] Regine WF, Winter KA, Abrams R, Safran H, Hoffman JP, Konski A, Benson AB, Macdonald JS, Rich TA, Willett CG (2011). Fluorouracil-based chemoradiation with either gemcitabine or fluorouracil chemotherapy after resection of pancreatic adenocarcinoma: 5-year analysis of the U.S. Intergroup/RTOG 9704 phase III trial. Ann Surg Oncol.

[25] Shimoda M, Kubota K, Shimizu T, Katoh M (2015). Randomized clinical trial of adjuvant chemotherapy with S-1 versus gemcitabine after pancreatic cancer resection. Br J Surg.

[26] Uesaka K, Boku N, Fukutomi A, Okamura Y, Konishi M, Matsumoto I, Kaneoka Y, Shimizu Y, Nakamori S, Sakamoto H, Morinaga S, Kainuma O, Imai K (2016). Adjuvant chemotherapy of S-1 versus gemcitabine for resected pancreatic cancer: a phase 3, open-label, randomised, non-inferiority trial (JASPAC 01). Lancet.

[27] Neoptolemos JP, Palmer D, Ghaneh P, Valle J, Cunningham D, Wadsley J, Meyer T, Anthoney A, Glimelius B, Falk S, Lind P, Izbicki J, Middleton G (2016). ESPAC-4: A Multicenter, International, Randomized Controlled Phase III Trial of Adjuvant Combination Chemotherapy of Gemcitabine (GEM) and Capecitabine (CAP), Versus Monotherapy Gemcitabine in Patients With Resected Pancreatic Ductal Adenocarcinoma. Pancreas.

[28] Sakuramoto S, Sasako M, Yamaguchi T, Kinoshita T, Fujii M, Nashimoto A, Furukawa H, Nakajima T, Ohashi Y, Imamura H, Higashino M, Yamamura Y, Kurita A (2007). Adjuvant chemotherapy for gastric cancer with S-1, an oral fluoropyrimidine. N Engl J Med.

[29] Mochizuki I, Takiuchi H, Ikejiri K, Nakamoto Y, Kinugasa Y, Takagane A, Endo T, Shinozaki H, Takii Y, Takahashi Y, Mochizuki H, Kotake K, Kameoka S (2012). Safety of UFT/LV and S-1 as adjuvant therapy for stage III colon cancer in phase III trial: ACTS-CC trial. Br J Cancer.

[30] Shirasaka T (2009). Development history and concept of an oral anticancer agent S-1 (TS-1): its clinical usefulness and future vistas. Jpn J Clin Oncol.

[31] Ueno H, Okusaka T, Ikeda M, Takezako Y, Morizane C (2005). An early phase II study of S-1 in patients with metastatic pancreatic cancer. Oncology.

[32] Chuah B, Goh BC, Lee SC, Soong R, Lau F, Mulay M, Dinolfo M, Lim SE, Soo R, Furuie T, Saito K, Zergebel C, Rosen LS (2011). Comparison of the pharmacokinetics and pharmacodynamics of S-1 between Caucasian and East Asian patients. Cancer Sci.

[33] MacBeth A, Law J, McGowan I, Norrie J, Thompson L, Wilson P (2015). Mellow Parenting: systematic review and meta-analysis of an intervention to promote sensitive parenting. Dev Med Child Neurol.

[34] Stocken DD, Büchler MW, Dervenis C, Bassi C, Jeekel H, Klinkenbijl JH, Bakkevold KE, Takada T, Amano H, Neoptolemos JP (2005). Meta-analysis of randomised adjuvant therapy trials for pancreatic cancer. Br J Cancer.

[35] Lumley T (2002). Network meta-analysis for indirect treatment comparisons. Stat Med.

